# Therapeutic efficacy of humanized monoclonal antibodies targeting dengue virus nonstructural protein 1 in the mouse model

**DOI:** 10.1371/journal.ppat.1010469

**Published:** 2022-04-29

**Authors:** Sen-Mao Tien, Po-Chun Chang, Yen-Chung Lai, Yung-Chun Chuang, Chin-Kai Tseng, Yu-San Kao, Hong-Jyun Huang, Yu-Peng Hsiao, Yi-Ling Liu, Hsing-Han Lin, Chien-Chou Chu, Miao-Huei Cheng, Tzong-Shiann Ho, Chih-Peng Chang, Shu-Fen Ko, Che-Piao Shen, Robert Anderson, Yee-Shin Lin, Shu-Wen Wan, Trai-Ming Yeh

**Affiliations:** 1 Department of Microbiology and Immunology, College of Medicine, National Cheng Kung University, Tainan, Taiwan; 2 Leadgene Biomedical, Inc. Tainan, Taiwan; 3 Institute of Basic Medical Sciences, College of Medicine, National Cheng Kung University, Tainan, Taiwan; 4 Department of Medical Laboratory Science and Biotechnology, College of Medicine, National Cheng Kung University, Tainan, Taiwan; 5 SIDSCO Biomedical Co., Ltd. Kaohsiung, Taiwan; 6 Department of Pediatrics, National Cheng Kung University Hospital, College of Medicine, National Cheng Kung University, Tainan, Taiwan; 7 Center of Infectious Disease and Signaling Research, National Cheng Kung University, Tainan, Taiwan; 8 Development Center for Biotechnology, Taipei, Taiwan; 9 Department of Microbiology and Immunology, Dalhousie University, Halifax, Canada; The University of Chicago, UNITED STATES

## Abstract

Dengue virus (DENV) which infects about 390 million people per year in tropical and subtropical areas manifests various disease symptoms, ranging from fever to life-threatening hemorrhage and even shock. To date, there is still no effective treatment for DENV disease, but only supportive care. DENV nonstructural protein 1 (NS1) has been shown to play a key role in disease pathogenesis. Recent studies have shown that anti-DENV NS1 antibody can provide disease protection by blocking the DENV-induced disruption of endothelial integrity. We previously demonstrated that anti-NS1 monoclonal antibody (mAb) protected mice from all four serotypes of DENV challenge. Here, we generated humanized anti-NS1 mAbs and transferred them to mice after DENV infection. The results showed that DENV-induced prolonged bleeding time and skin hemorrhage were reduced, even several days after DENV challenge. Mechanistic studies showed the ability of humanized anti-NS1 mAbs to inhibit NS1-induced vascular hyperpermeability and to elicit Fcγ-dependent complement-mediated cytolysis as well as antibody-dependent cellular cytotoxicity of cells infected with four serotypes of DENV. These results highlight humanized anti-NS1 mAb as a potential therapeutic agent in DENV infection.

## Author summary

DENV comprising four serotypes has a complicated pathogenesis and remains an unresolved global health problem. To date, supportive therapy is the mainstay for treatment of dengue patients. Despite a licensed Sanofi vaccine and ongoing clinical trials, more effective vaccines and/or licensed therapeutic drugs are required. Therapeutic mAbs are a potential tool to treat many epidemic diseases because of their high target specificity. Humanized anti-NS1 mAbs can recognize the NS1 from all four serotypes of DENV without danger of inducing ADE. In the DENV infection mouse model, we demonstrate that humanized NS1 mAbs have therapeutic benefits such as reducing DENV-induced prolonged bleeding time and skin hemorrhage. In vitro mechanistic studies showed a reduction of NS1-induced vascular permeability and an increase in cytolysis of DENV-infected cells. Our results showed that humanized anti-NS1 mAbs show strong potential for development toward clinical use.

## Introduction

Dengue virus (DENV) poses a threat to more than half of the world population. There are an estimated 390 million infections annually, of which 96 million cases show apparent manifestations [1]. Severe DENV infection may result in dengue hemorrhagic fever or dengue shock syndrome (DHF/DSS) [2,3]. The need for safe and effective vaccines and therapeutic agents is crucial to control dengue disease [4,5].

To date, there is a lack of approved therapeutic antibodies or antiviral drugs for DENV infection. Antibodies against envelope (E) protein have received much attention due to their ability to provide neutralizing protection against DENV [6–9]. However, in addition to the beneficial neutralization effect of E-specific antibodies, nonneutralizing or subneutralizing antibodies against E may result in harmful antibody-dependent enhancement (ADE) of infection [10–13] leading to more severe disease and even death [14]. In recent years, nonstructural protein 1 (NS1) has emerged as an attractive candidate for antiviral or vaccine strategies against DENV [5,14–17]. NS1 can be expressed as a dimer on the surface of infected cells and is also secreted as a soluble hexamer. Levels and duration of NS1 in patient sera are associated with disease severity, in particular vascular leakage [18–24]. Secreted NS1 can act via TLR4 as a viral toxin to cause the release of proinflammatory cytokines and/or disrupt the endothelial glycocalyx, which may lead to hyperpermeability and toxic shock syndrome [25–31]. NS1 can also activate platelets via TLR4, leading to thrombocytopenia and hemorrhage [32]. Moreover, NS1 can trigger peripheral blood mononuclear cells (PBMC) to produce proinflammatory cytokines such as IL-6 and TNF-α [33].

Recent studies have demonstrated that broad-spectrum anti-flavivirus NS1 monoclonal antibodies (mAbs) can inhibit DENV-associated endothelial dysfunction and provide protection in mice [14,15]. Using the DENV-mouse model, our previous studies showed that passive administration with NS1 polyclonal antibodies or mAbs reduced DENV-induced prolonged bleeding time and hemorrhage [34–36]. In this study, we have generated humanized mAbs against DENV NS1 and demonstrated their protective effects in mice. Furthermore, in vitro mechanistic studies revealed that blockage of endothelial permeability, induction of complement-dependent cytolysis (CDC) and antibody-dependent cellular cytotoxicity (ADCC) all contribute to the effectiveness of humanized anti-NS1 mAbs. The results of our study thus provide support for humanized antibodies as a therapeutic candidate in severe DENV infection.

## Results

### Generation and characterization of humanized mAbs 2E8 and 33D2

**Fig 1:**
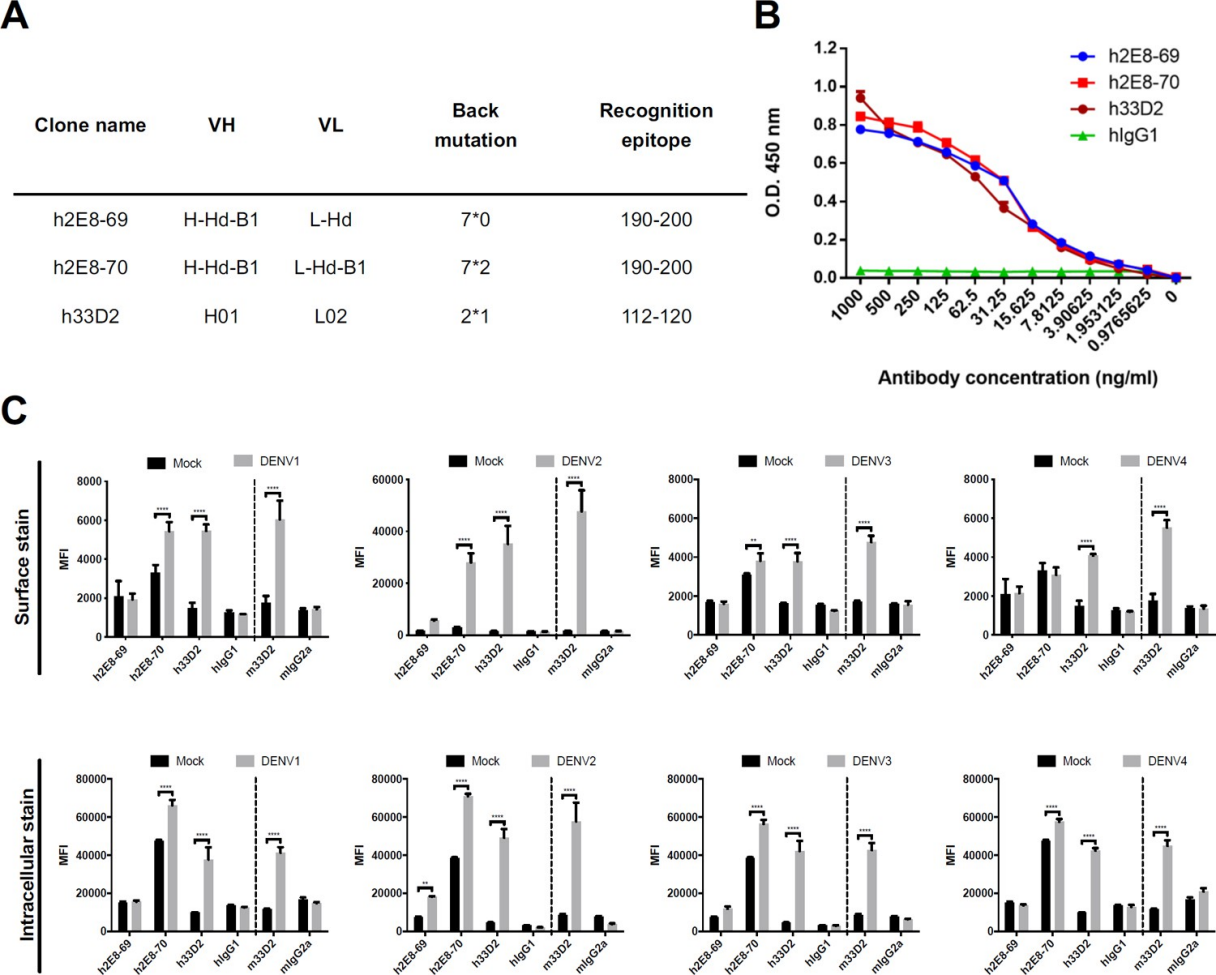
Comparison and characterization of three humanized mAb candidates. (**A**) The details of h2E8-69, h2E8-70 and h33D2 are shown. The numbers of back mutation of each humanized mAb are shown as heavy chain mutation number*light chain mutation number. (**B**) The binding activity of each humanized mAb including h2E8-69, h2E8-70, h33D2 and isotype control hIgG1 to full length NS1 was determined by ELISA. (**C**) The binding ability of h2E8-69, h2E8-70, h33D2, isotype control hIgG1, mouse 33D2 (m33D2), and isotype control mouse IgG2a (mIgG2a) to DENV-infected cells were analyzed by flow cytometery. Huh7 cells were infected by four different DENV serotypes (MOI = 2: DENV2 and DENV4; MOI = 5: DENV1 and DENV3) for 48 h. Non-fixed (upper panel) and paraformaldehyde-fixed (lowed panel) Huh7 cells were incubated with mouse anti-NS1 mAb m33D2, humanized anti-NS1 mAbs h33D2, h2E8-69, h2E8-70 as well as mouse isotype control IgG2a or human isotype control IgG1, followed by FITC-conjucated anti-mouse/human IgG and analyzed by flow cytometry. All data are presented as the averages of triplicate cultures ± S.D. **: p < 0.01, ****: p < 0.0001. Statistical significance was based on two-way ANOVA. MFI: mean fluorescence intensity.

We previously reported that murine mAb 2E8 which recognizes DENV NS1 amino acids 190–200 [35] and mAb 33D2 which recognizes amino acids 112–120 [36] provided protection against four serotypes of DENV. In the current study, the humanized mAbs 2E8 (h2E8) and 33D2 (h33D2) were created by grafting the antigen-binding loops, known as complementarity-determining regions (CDRs), from the mouse mAbs into a human IgG1. Based on their lower back mutation and/or the NS1-binding ability, we further tested two h2E8 candidates (clones 69 and 70) and one h33D2 candidate (Fig 1A). ELISA analysis confirmed that h2E8-69, h2E8-70 and h33D2 were recognized by anti-human IgG (S1B Fig), but not by anti-mouse IgG (S1A Fig). Mouse mAbs 2E8, 33D2, isotype control mouse IgG1 and IgG2a were used as the controls (S1A and S1B Fig). We compared the binding ability of these humanized mAbs against NS1 as determined by ELISA. The results showed that h2E8-69, h2E8-70 and h33D2 have similar binding activity to NS1 at different dilutions (Fig 1B).

The binding ability of various humanized mAbs including h2E8-69, h2E8-70, h33D2, isotype control human IgG1 (hIgG1), mouse 33D2 (m33D2), and isotype control mouse IgG2a (mIgG2a) to Huh7 cells infected with four different DENV serotypes were analyzed by flow cytometry. Mouse 33D2 mAbs that can recognize NS1 from all serotypes of DENV were used as positive controls [36]. The results showed that h33D2 mAbs recognized surface and intracellular NS1 on four serotypes of DENV-infected cells. In addition, the NS1 binding ability of h33D2 to four serotypes of DENV is superior to that of h2E8-70 and h2E8-69 (Fig 1C).

We also determined the stability of humanized mAbs at body temperature 37°C. Using a Nanodrop 2000 spectrophotometer to measure UV light absorbance at 280 nm, the concentrations of h2E8-69, h2E8-70, h33D2, hIgG1 as well as m2E8, m33D2, mIgG1 and mIgG2a were determined at 7, 14, and 30 days from an original concentration of 200 μg/ml. The final antibody concentrations after different time intervals are shown (S2A Fig). There was only a slight decline after 30 days, suggesting that the humanized mAb candidates are all stable at 37°C. We then investigated the binding ability of humanized mAbs to target antigen, NS1, as an indicator of stability. After 7, 14, and 30 days at 37°C, the binding ability of h2E8-69, h2E8-70 and h33D2 to full length NS1 was examined by ELISA. The results demonstrated that in contrast to isotype control hIgG1, h2E8-69, h2E8-70 and h33D2 bound to full length NS1, and the binding abilities of these humanized mAbs at different time points were similar (S2B Fig).

To rule out the possibility of ADE mediated by these humanized mAbs, DENV2 was mixed with PBS (DENV2 only group), control mouse IgG (cmIgG), h2E8-69, h2E8-70, h33D2, hIgG1, and anti-prM mAb 70.21 as a positive control, respectively, and then inoculated into U937 cells. The viral titers were determined after 48 h. As expected, the group pretreated with anti-prM mAb 70.21 showed an increased viral titer compared to DENV2 only group. In contrast, the viral titers of DENV-infected U937 cells pretreated with h2E8-69, h2E8-70, or h33D2 were similar to or even lower than with DENV2 only, confirming that these humanized mAbs do not pose a risk of ADE (S3 Fig).

### Administration of humanized mAbs 2E8 and 33D2 reduces DENV-induced prolonged bleeding time and skin hemorrhage in mice

**Fig 2:**
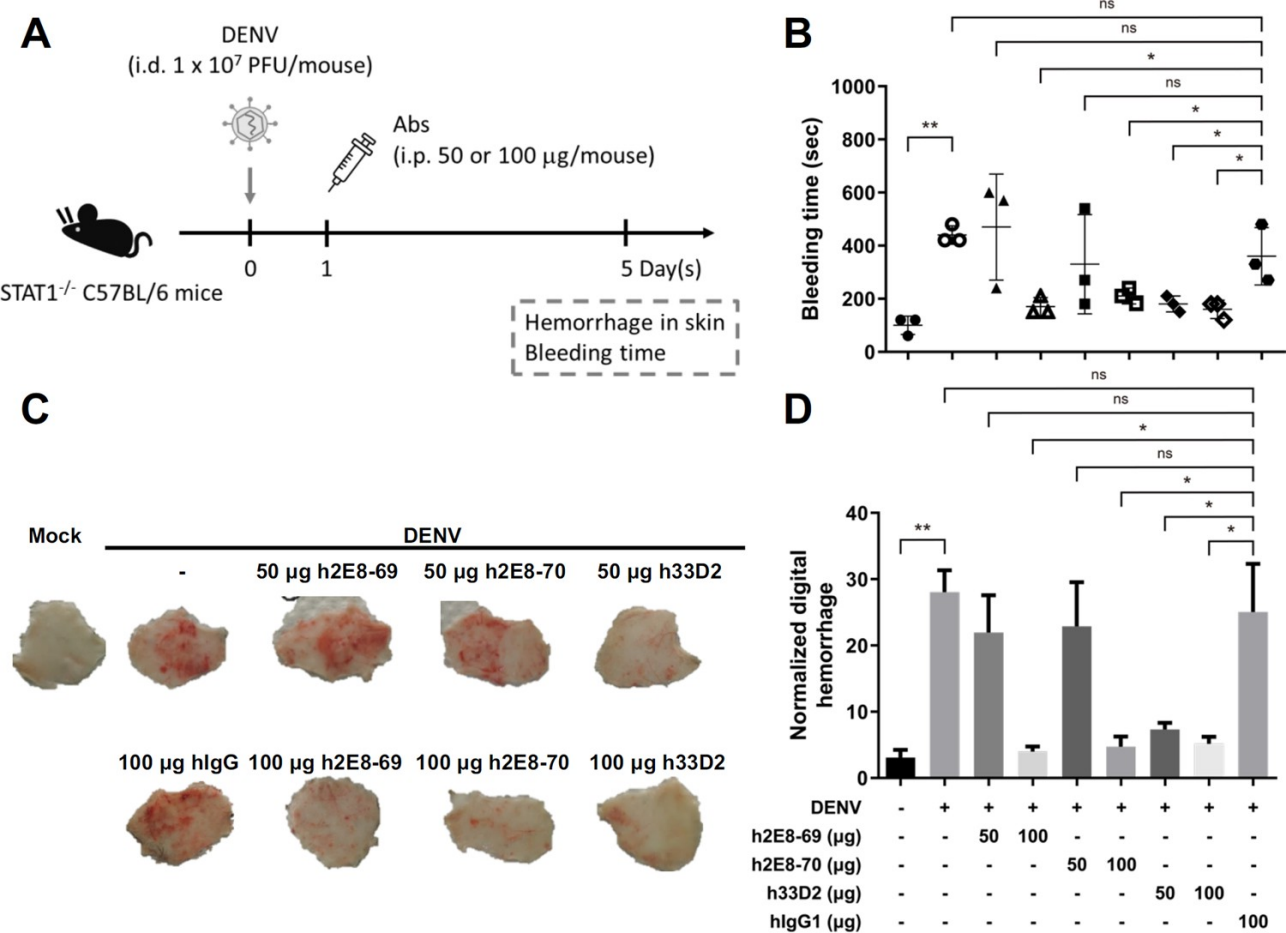
Administration of humanized anti-NS1 mAbs 2E8 and 33D2 can shorten DENV-induced prolonged bleeding time and reduce skin hemorrhage. (**A**) The schematic model illustrates the therapeutic treatment of mice. 1 × 10^7^ PFU/mouse DENV2-454009A or C6/36 control medium were inoculated i.d. into the upper back of *STAT1*^*-/-*^ mice. The mAbs h2E8-69, h2E8-70, h33D2, or isotype control hIgG1 (50 or 100 μg/mouse) were injected i.p. one day after virus challenge. (**B**) The tail bleeding time was determined on 5 d.p.i. (n = 3 for each group) (C) Mice were sacrificed on day 5, and the fresh skin samples were obtained from the upper back to observe local skin hemorrhage. (**D**) The degree of hemorrhage was quantified by ImageJ software analysis as described in the methods. (n = 4 for 100 μg hIgG1 and n = 5 for other groups) *: p < 0.05, **: p < 0.01, ns indicates not significant, as analyzed by one-way ANOVA and followed by Tukey’s multiple comparison test.

To evaluate the therapeutic effect provided by anti-NS1 mAbs h2E8 and h33D2 in vivo, we used the DENV infection hemorrhage mouse model with modifications [37–39]. *STAT1*^-/-^ mice were intradermally (i.d.) inoculated with 1 × 10^7^ PFU/mouse of DENV2-454009A at four sites on the upper back. Mice were then administered intraperitoneally (i.p.) with h2E8 or h33D2 at 1 day-post-infection (d.p.i). This time point was chosen because of the known peak level of secreted NS1 at 12 to 24 h post-infection [36]. At 5 d.p.i., the skin hemorrhage and mouse tail bleeding time were determined (Fig 2A). The results showed that treatment with 100 μg/mouse h2E8-69, h2E8-70 and h33D2 all caused a reduction of DENV-induced prolonged bleeding time (Fig 2B) and skin hemorrhage (Fig 2C and 2D) compared to mice treated with isotype control hIgG1. Furthermore, passive administration of 50 μg/mouse h33D2 at 1 d.p.i. also provided similar therapeutic effect as 100 μg/mouse h33D2, whereas 50 μg/mouse h2E8-69 and h2E8-70 did not cause significant reduction in DENV-induced prolonged bleeding time (Fig 2B) and skin hemorrhage (Fig 2C and 2D). Mice inoculated with i.d. culture medium (Mock) and with i.p. PBS (DENV2 only) served as controls. Since anti-E antibodies have been recognized to provide neutralizing protection against DENV [6–9], we compared the therapeutic effect provided by anti-NS1 mAb m33D2 and anti-E mAb m137-22 in this mouse model. Results showed that both m33D2 and m137-22 cause significant reduction in DENV-induced prolonged bleeding time, although m33D2 provided better protection than m137-22 (S4 Fig).

### Reduction of viremia and soluble NS1 by humanized mAb 33D2

**Fig 3:**
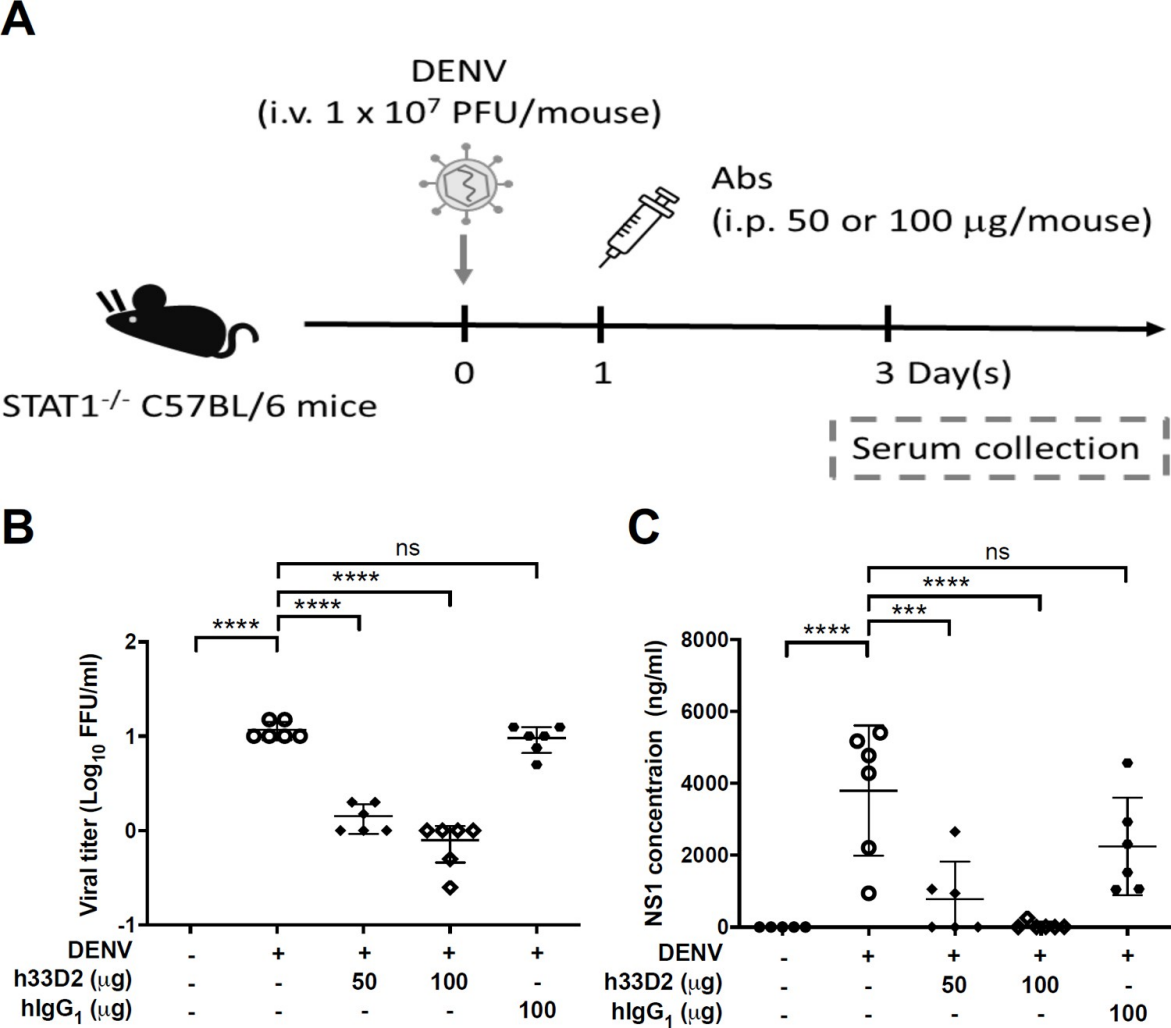
Passive transfer of humanized anti-NS1 mAb 33D2 into DENV-inoculated mice decreases viral titer and NS1 level in mouse sera. (**A**) Schematic diagram for the therapeutic treatment of mice. The h33D2 (50 or 100 μg/mouse) or hIgG1 (100 μg/mouse) were injected i.p. one day after virus challenge. 1 × 10^7^ PFU/mouse DENV2-454009A or C6/36 control medium were inoculated i.v. into *STAT1*^*-/-*^ mice. (**B**) Viremia at 3 d.p.i. was analyzed by fluorescent focus assay. (**C**) Mouse sera were collected at 3 d.p.i., and the secreted NS1 levels were analyzed by NS1 quantitative ELISA. (n = 5 for mock and n = 6 for other groups) ***: p < 0.001, ****: p < 0.0001, ns indicates not significant as compared with DENV alone group and based on one-way ANOVA followed by Dunnett’s multiple comparison test.

We further investigated the potential ability of h33D2 to reduce viral titer and soluble NS1 level. Mice were inoculated intravenously (i.v.) with DENV2 strain 454009A, which can actively replicate in *STAT1*^-/-^ mice. We then administered mice i.p. with h33D2 or hIgG1 at 1 d.p.i. and collected blood samples at 3 d.p.i. (Fig 3A). The results showed that h33D2 treatment reduced the viral titer (Fig 3B). Because NS1 is released from the infected cells during DENV replication, we also determined the NS1 levels in the mouse sera. The results showed that serum levels of NS1 were also reduced by the administration of 50 or 100 μg/mouse of h33D2 (Fig 3C).

### Dose response and time kinetics of humanized mAb 33D2 on the protective efficacy in the DENV-infected mouse model

**Fig 4:**
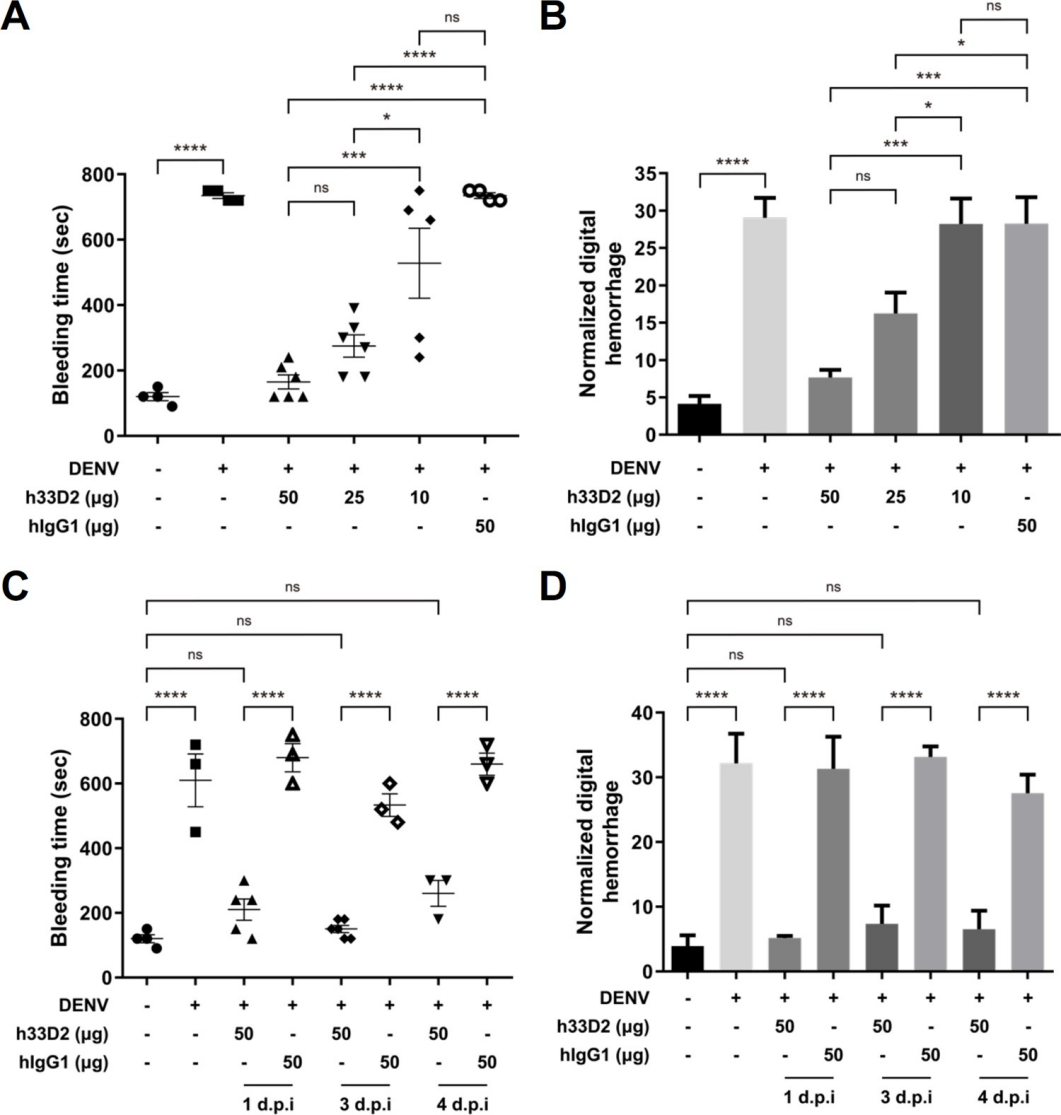
Dose response and time kinetics of bleeding time and hemorrhage following passive transfer of humanized anti-NS1 mAb 33D2 into DENV-infected mice. Mice were inoculated with 1 × 10^7^ PFU/mouse DENV2-454009A or C6/36 control medium i.d. into the upper back of *STAT1*^*-/-*^ mice. (**A, B**) The mAb h33D2 (10, 25, or 50 μg/mouse) or 50 μg/mouse isotype control hIgG1 was injected i.p. one day after DENV challenge. (**A**) The tail bleeding time was determined on 5 d.p.i. (n = 6 for 50 μg and 25 μg h33D2 groups, n = 5 for 10 μg h33D2 group, and n = 4 for other groups). (**B**) The skin hemorrhage was determined on 5 d.p.i. (n = 4 for mock and n = 5 for other groups). (**C, D**) The mAb h33D2 or isotype control hIgG1 (50 μg/mouse) was injected i.p. 1, 3 or 4 days after DENV challenge. (**C**) The tail bleeding time was determined on 5 d.p.i. (n = 4 for mock group, n = 5 for h33D2-treated on 1 d.p.i. group, n = 6 for h33D2-treated on 3 d.p.i. group, and n = 3 for other groups) (**D**) The skin hemorrhage was determined on 5 d.p.i. (n = 4 for each group) *: p < 0.05, ***: p < 0.001, ****: p < 0.0001, ns indicates not significant. Statistical significance was based on one-way ANOVA followed by Tukey’s multiple comparison test.

Since passive administration of 100 and 50 μg/mouse h33D2 at 1 d.p.i. provided similar therapeutic effects, we further investigated whether lower doses provided protection in mice. The results showed a dose response effect of protection in the DENV-induced prolonged bleeding time (Fig 4A) and skin hemorrhage (Fig 4B), by which 50 μg/mouse h33D2 conferred nearly complete protection, 25 μg/mouse h33D2 conferred approximately 50% protection, and 10 μg/mouse h33D2 did not confer protection.

We next evaluated the protective effect when the h33D2 administration time was postponed from day 1 to day 3 or day 4 post-infection. The results showed that treatment with 50 μg/mouse h33D2 at 3 or 4 d.p.i. still reduced DENV-induced prolonged bleeding time (Fig 4C) and skin hemorrhage (Fig 4D). These results indicate that h33D2 provides significant protective effects against DENV infection over a range of time points.

### The modes of action of humanized mAb 33D2 as shown by in vitro mechanistic studies

To gain insights into the protective effects provided by h33D2, we considered three potential mechanisms namely vascular permeability, complement-dependent cytolysis (CDC) and antibody-dependent cellular cytotoxicity (ADCC).

**Fig 5:**
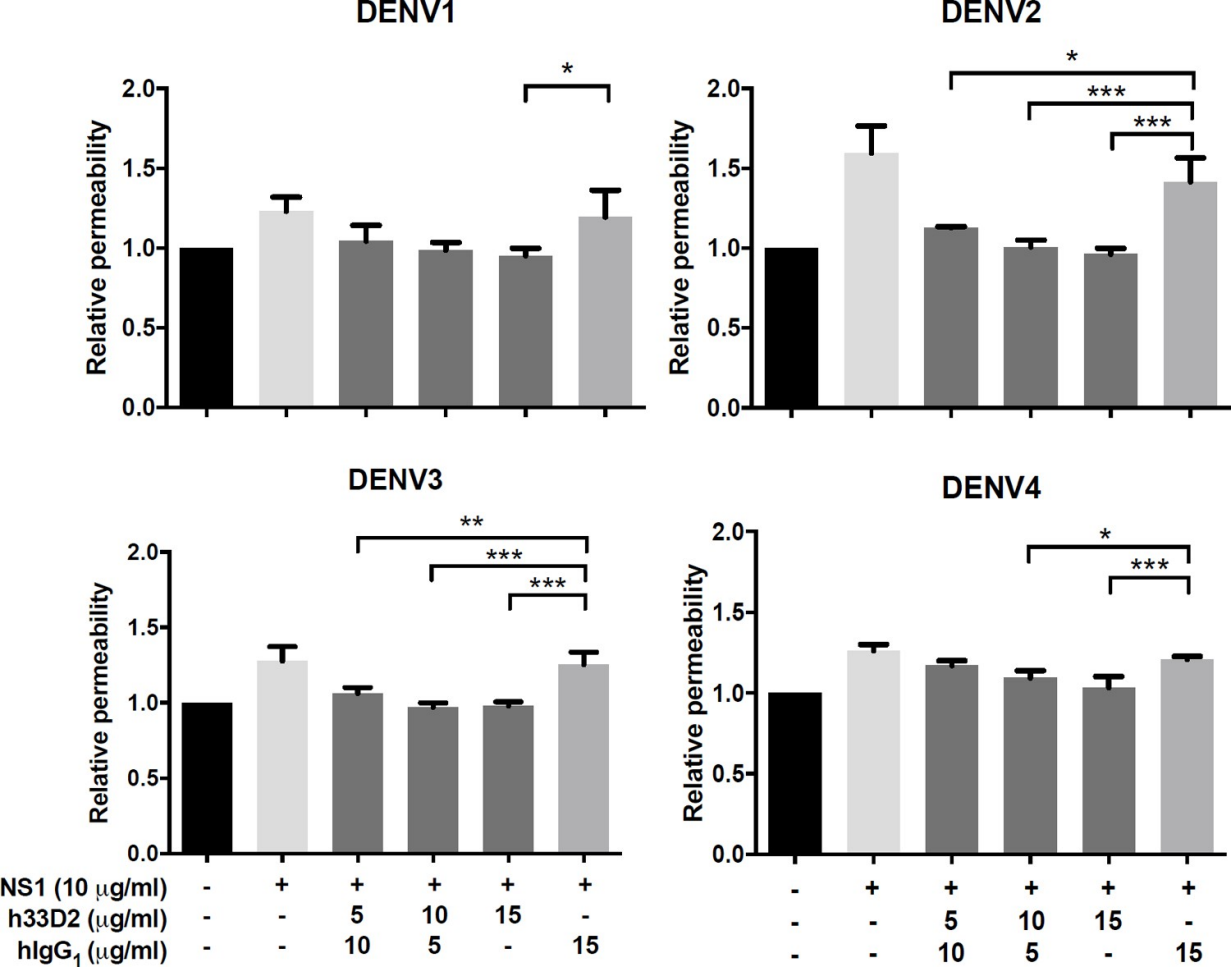
Humanized anti-NS1 mAb 33D2 can reduce NS1-induced endothelial permeability. Isotype control hIgG1 was combined with different doses of h33D2 at 15 μg/ml final antibody concentration. DENV NS1 from serotypes 1, 2, 3 and 4 was used at 10 μg/ml of final concentration. DENV1-4 NS1-induced HMEC-1 cell hyperpermeability was determined as described in the methods. All data are presented as the averages of triplicate cultures ± S.D. *: p < 0.05, **: p < 0.01,***: p < 0.001, as compared with hIgG1 group and analyzed by one-way ANOVA followed by Dunnett’s multiple comparison test.

NS1-induced vascular permeability increase is a hallmark for DENV disease [25–31]. We used isotype control combined with different doses of h33D2 at a 15 μg/ml final antibody concentration. DENV NS1 from serotypes 1, 2, 3 and 4 was used at a final concentration of 10 μg/ml. The hyperpermeability induced by DENV1-4 NS1 is similar with which induced by 1000 pg/ml TNF-α treatment (S5 Fig). The results showed that treatment with h33D2 caused a dose-dependent inhibition of DENV1-4 NS1-induced HMEC-1 cell hyperpermeability as compared with control hIgG1 (Fig 5).

An L234A, L235A, P329G (LALAPG) variant in the Fc region of IgG eliminates complement binding and fixation as well as Fcγ-dependent cytotoxicity in human IgG1 [40,41]. The LALAPG variant and its wild-type counterparts had comparable binding activities to full-length DENV NS1 (S6A Fig). Moreover, we monitored the levels of h33D2 and h33D2-LALAPG in mouse sera at 4 h, 2 days and 4 days post-administration. Results showed that there was similar patten of antibody clearance in h33D2- and h33D2-LALAPG-treated group (S6B Fig). Th results suggested that these substitutions do not alter the binding activity to NS1 protein and clearance/ decline rate of antibodies in vivo.

**Fig 6:**
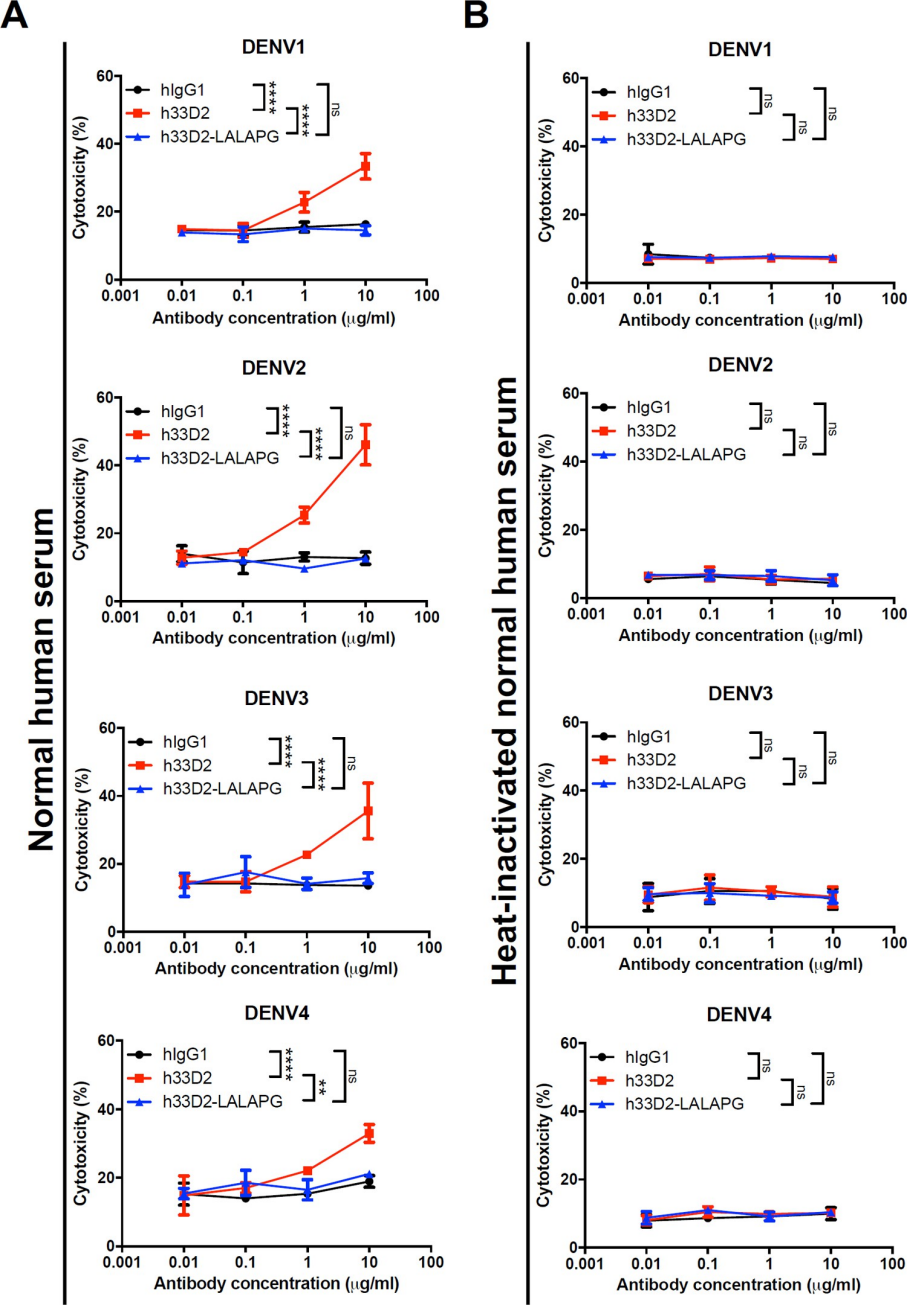
Fc-dependence of complement-mediated lysis of DENV-infected cells by humanized anti-NS1 mAb 33D2. Huh-7 cells were infected with DENV1-4 then using isotype control hIgG1, h33D2, and h33D2-LALAPG at 0.01, 0.1, 1, and 10 μg/ml final concentrations to analyze ability to elicit CDC by using normal human serum (NHS) (**A**) or heat-inactivated NHS (**B**). The LALAPG variant preparation and the CDC assay were described in the methods. All data are presented as the averages of triplicate cultures ± S.D. **: p < 0.01, ****: p < 0.0001, ns indicates not significant. Statistical significance was based on two-way ANOVA followed by Tukey’s multiple comparison test.

To further confirm the Fc-dependence on CDC activity, we used isotype control hIgG1, h33D2, and h33D2-LALAPG at 0.01, 0.1, 1, and 10 μg/ml final concentrations. Cytolysis was measured by the release of lactate dehydrogenase (LDH). We found that h33D2, but not isotype control hIgG1 and h33D2-LALAPG, could induce CDC in a dose-dependent manner (Fig 6A). Heat-inactivated normal human serum (NHS) was used as a control to verify the complement-dependence of the cytotoxic effect (Fig 6B).

**Fig 7:**
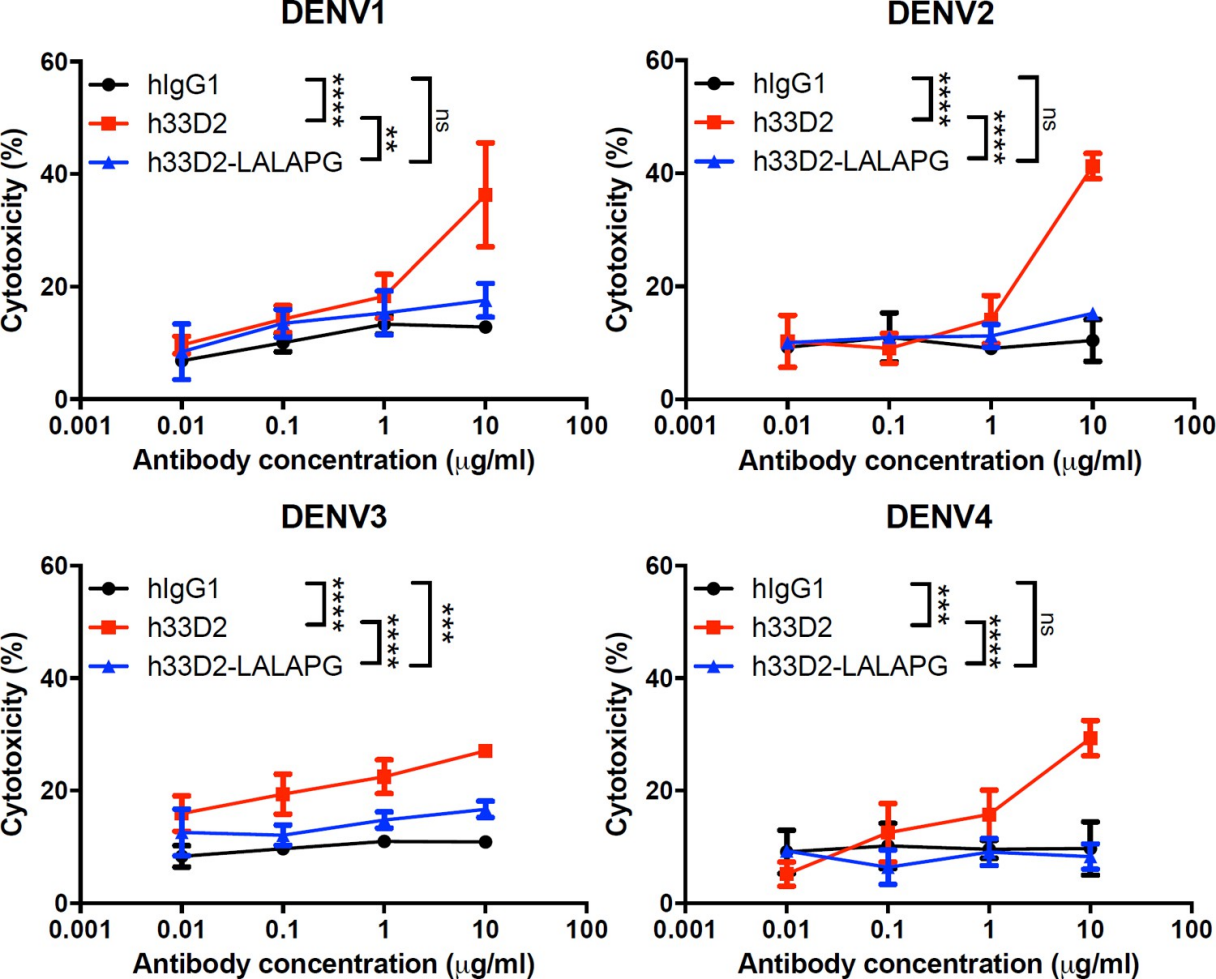
Fc-dependence of antibody-dependent cellular cytotoxicity of DENV-infected cells by humanized anti-NS1 mAb 33D2. Huh-7 cells were infected with DENV1-4 then using isotype control hIgG1, h33D2, and h33D2-LALAPG at 0.01, 0.1, 1, and 10 μg/ml final concentrations to analyze ability to elicit ADCC by using NK cells. The LALAPG variant preparation and the ADCCassay were described in the methods. All data are presented as the averages of triplicate cultures ± S.D. **: p < 0.01, ***: p < 0.001, ****: p < 0.0001, ns indicates not significant. Statistical significance was based on two-way ANOVA followed by Tukey’s multiple comparison test.

**Fig 8:**
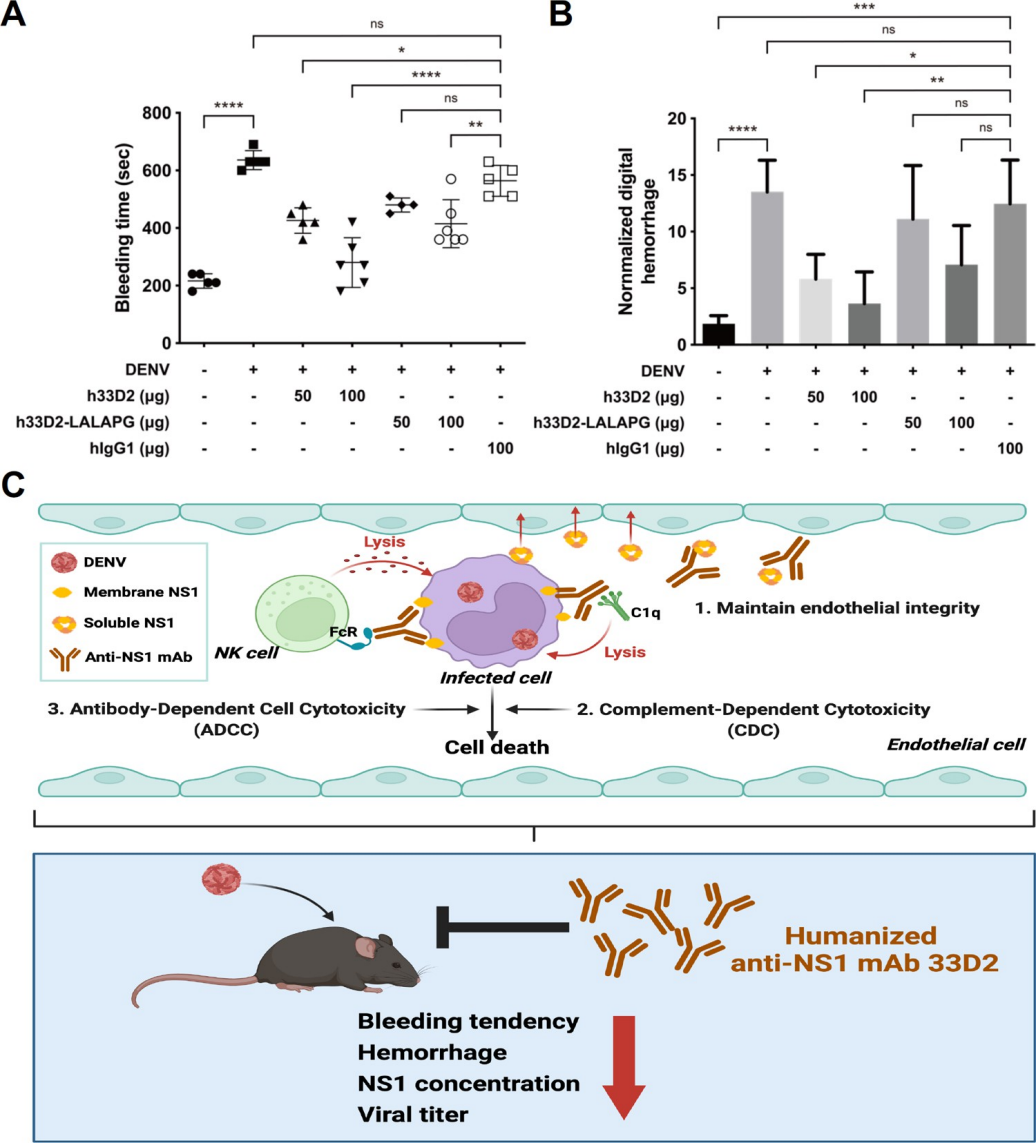
Effects of humanized anti-NS1 mAb 33D2 and 33D2-LALAPG on DENV-induced prolonged bleeding time and skin hemorrhage. 1 × 10^7^ PFU/mouse DENV2-454009A or C6/36 control medium were inoculated i.d. into the upper back of *STAT1*^*-/-*^ mice. The mAbs h33D2, h33D2-LALAPG or isotype control hIgG1 (50 or 100 μg/mouse) were injected i.p. one day after virus challenge. (**A**) Tail bleeding time was determined on 5 d.p.i. (**B**) The degree of hemorrhage was quantified by ImageJ software analysis as described in the methods. (n = 6 for the 100 μg h33D2-treated and h33D2-LALAPG-treated groups, n = 4 for the 50 μg h33D2-LALAPG-treated group, and n = 5 for other groups) *: p < 0.05, **: p < 0.01, ***: p < 0.001, ****: p < 0.0001, ns indicates not significant. Statistical significance was based on one-way ANOVA followed by Tukey’s multiple comparison test. (**C**) The schematic model shows that anti-NS1 mAbs block NS1-induced hyperpermeability to maintain endothelial integrity as well as trigger complement-mediated cytotoxicity (CDC) and antibody-dependent cellular cytotoxicity (ADCC) to cause infected cell death. Hence, in the mouse model, anti-NS1 mAbs not only reduce DENV-induced prolonged bleeding time and hemorrhage, but also reduce serum viral titer and soluble NS1 level. This figure is created with Biorender.com.

ADCC is largely mediated by NK cells through the CD16 (FcγRIII) receptor that binds the Fc portion of IgG triggering the lysis of target cells. In the ADCC experiment, we used isotype control hIgG1, h33D2, and h33D2-LALAPG at 0.01, 0.1, 1, and 10 μg/ml final concentrations and determined Huh-7 cell death induced by NK cells. The E (Effector cells)/T (Target cells) ratio was 25:1. The results showed that h33D2 at 10 μg/ml caused significant NK cell cytotoxic effect against all four serotypes of DENV-infected Huh-7 cells, whereas h33D2-LALAPG did not cause NK cell cytotoxic effect compared with isotype control hIgG1 (Fig 7). We further found that h33D2-LALAPG provided less protection than h33D2 in the DENV-induced prolonged bleeding time (Fig 8A) and skin hemorrhage (Fig 8B). Therefore, a functional Fc region is important for the therapeutic effects of h33D2 in mice. When we administered h33D2-LALAPG to mice on day 4 and determined the bleeding time on day 5, results showed that, similar to h33D2, h33D2-LALAPG can also reduce DENV-induced prolonged bleeding time at the later time point (S7 Fig). Therefore, in addition to Fc-mediated CDC and ADCC, other mechanisms may also be involved in the protective effects.

## Discussion

There is still an urgent need for effective control agents for DENV. Therapeutic antibodies may be considered as a protective option due to their ability to be engineered to improve their efficacy and their high tolerance by humans. Accordingly, therapeutic antibodies are being increasingly used for the treatment of various infectious diseases [6]. A number of groups have reported DENV-specific antibodies against E protein [7–9,42], but the risk of ADE must be carefully evaluated. The DENV NS1 protein, which is not a virion-associated protein, represents an attractive target for antiviral or vaccine approaches without the danger of ADE. NS1 exerts several pathogenic effects during the critical phase of dengue disease. NS1 disrupts endothelial cell integrity causing hyperpermeability and activates platelets contributing to thrombocytopenia [25–32]. Moreover, NS1 binds to thrombin/prothrombin which perturbs the coagulation system [43]. Furthermore, NS1-induced endothelial hyperpermeability can be reversed by anti-NS1 mAb treatment, which can thus be considered as a therapeutic candidate [14,15,35,36]. Nevertheless, a recent study showed that neutralization of circulating sNS1 through antibody transfer or active NS1 immunization did not confer protection against disease induced by a DENV2 strain D2Y98P [44]. Therefore, the therapeutic effects on different viral strains need to be further evaluated and considered.

Previous studies on DENV2 and DENV3 infected pediatric patients with primary and secondary infection showed that the most immunodominant regions of NS1 were those represented by amino acids 101–130 and 296–325 [45]. Another study [46] in pediatric patients with acute secondary DENV1 showed somewhat overlapping immunodominant regions (amino acids 121–142 and 291–307) and DENV2 (amino acids 297–313), although they also found most of the antibodies were directed at different regions of NS1. In our study, we used mAb 2E8 which recognizes DENV NS1 amino acids 190–200 and mAb 33D2 which recognizes wing domain amino acids 112–120. The epitope recognized by mAb 33D2 was similar to that reported by Hertz et al. [45]. The mAb 1G5.3 used in a recent study [15] with a broad protective effect to flavivirus NS1 recognizes an epitope at the distal end of the β-ladder domain of C-terminal fragment. The mAb 2B7 in another study [14] also recognizes the β-ladder domain of NS1 (amino acids 260–316 and 288–344), yet 2B7 may create indirect steric hindrance of the wing domain to interfere with the binding of NS1 wing domain to the cell surface. Therefore, antibodies directed to either the β-ladder or wing domain are likely to interfere with NS1-mediated endothelial dysfunction [14].

An important safety concern of anti-NS1 antibodies is autoreactivity. Previous studies showed that anti-DENV NS1 antibodies cross-reacted with clotting factors, platelets and endothelial cells, causing their dysfunction and endothelial cell damage [47–51]. The C-terminal region of the DENV NS1 protein contains cross-reactive epitopes shared with self-antigens, mostly located at the C-terminal end (amino acids 311–330) [52–54]. Our recent studies showed that mouse anti-NS1 mAbs 2E8 and 33D2 do not recognize the C-terminal NS1 of DENV or host proteins such as prothrombin/thrombin, platelets, and endothelial cells [35,36]. We have therefore generated humanized mAbs 2E8 and 33D2 and confirmed their therapeutic effects in the mouse model and mode of actions in vitro in the current study. Anti-NS1 mAbs have several advantages, for instance, these mAbs can provide protection without the risk of ADE. Moreover, the DENV NS1 is conserved to about 79% across the four serotypes [45]. Anti-NS1 antibodies can decrease NS1-induced pathogenesis in the critical disease period, and anti-prM/M or anti-E antibodies are effective in the viremic phase [5]. Therefore, our humanized anti-DENV NS1 mAbs could find utility in the critical phase for reducing pathogenesis and may also be considered in combination with anti-E mAbs in the early viremic phase.

As for the possible mechanisms of action of therapeutic anti-NS1 mAbs, these mAbs can suppress viral propagation by CDC of DENV-infected cells since NS1 is anchored on the membrane of infected cells. Another possible mechanism for inhibiting virus propagation is ADCC. Previous studies demonstrated that infection with DENV can elicit antibodies that are capable of promoting ADCC via NK cell activation to reduce the severity of disease [55–57]. Our data using the LALAPG variant showed that Fcγ-dependent CDC and ADCC can be mediated by h33D2 but not h33D2-LALAPG (Figs 6 and 7). However, h33D2-LALAPG can reduce but not completely inhibit DENV-induced prolonged bleeding time and hemorrhage (Fig 8A and 8B), suggesting that CDC and ADCC play partial but not complete roles in the protective effects. We gave mice h33D2 at different time points and found that administration of h33D2 at 1, 3, or 4 d.p.i. can reduce DENV-associated prolonged bleeding time and hemorrhage severity in local skin tissue. In other words, h33D2 effectively protected mice against DENV pathogenic effects even when a single dose was administered as late as 24–48 h prior to severe bleeding. We further administered h33D2-LALAPG to mice on day 4 and determined the bleeding time on day 5. Results showed that similar to h33D2, h33D2-LALAPG can also reduce DENV-induced prolonged bleeding time at the later administration time (S7 Fig). Therefore, in addition to Fc-mediated CDC and ADCC, other mechanisms may also be involved in the protective effects, such as direct effects on NS1-induced vascular hyperpermeability (Fig 5) or on changes in coagulation activity and inflammatory cytokine production which need to be further confirmed. Our findings are in agreement with a recent report [15] that anti-NS1 mAbs may provide protection through the direct inhibition of NS1 toxicity as well as Fc-dependent mechanisms such as ADCC. The schematic model of this study is summarized in Fig 8C showing the in vitro CDC and ADCC as well as maintaining endothelial integrity mediated by humanized mAb 33D2. Furthermore, the protection provided by humanized mAb 33D2 is demonstrated in DENV-challenged mice.

In conclusion, our results suggest that humanized anti-NS1 mAbs have potential for therapeutic application against all four serotypes of DENV. In addition to DENV, NS1-mediated endothelial dysfunction has been observed for various flaviviruses including Zika virus, West Nile virus, Japanese encephalitis virus, and yellow fever virus [58,59], and importantly, broadly protective mAbs against flaviviral NS1 have recently been reported [14,15]. Whether h33D2 and h2E8 can recognize other flaviviral NS1 and provide protection remains to be determined.

## Material and methods

### Ethics statement

All animal studies were performed in strict accordance with the Experimental Animal Committee of National Cheng Kung University (NCKU) and were approved by the Institutional Animal Care and Use Committee (IACUC) of NCKU under the number IACUC 106272 and 109309.

All research involving healthy adult donors was approved by the NCKU Hospital Institutional Review Board (IRB # A-ER-109-535). Informed written consent was obtained from volunteers following the human experimentation guidelines of the Institutional Review Board of NCKU Hospital.

### Mice

*STAT1*-deficient C57BL/6 (*STAT1*^-/-^ B6) mice were bred at the National Laboratory Animal Center (Tainan) and were maintained on standard laboratory food and water. The 4- to 8-week-old progeny were used in the therapeutic animal model studies reported here. The experiments were approved by the IACUC of NCKU.

### Virus cultures

DENV1 strain 8700828, DENV2 strain 454009A, DENV3 strain 8700829, and DENV4 strain 59201818 were maintained in C6/36 cells. In brief, monolayers of C6/36 cells were incubated with DENV at a multiplicity of infection (MOI) of 1 and then incubated in 5% CO_2_ at 28°C for 4 to 7 days. The culture medium was harvested, and cell debris was removed by centrifugation at 1200 rpm for 10 min. To increase the viral titer, the virus supernatant was collected and concentrated by centrifugation at 3000 rpm for 20 min at 4°C using Amicon Ultra Centrifugal Filters (100K) (Millipore). Virus stocks were stored below −70°C until use. Virus titer was determined by plaque forming assay using BHK-21 cells.

### Cell cultures

C6/36 and Huh-7 cells were cultured in Dulbecco’s modified Eagles medium (DMEM) (Thermo Fisher Scientific) containing antibiotics and 10% fetal bovine serum (FBS). BHK-21 cells were grown in Roswell Park Memorial Institute (RPMI) medium (Thermo Fisher Scientific) containing 5% FBS. U937 cells were cultured in RPMI-1640 medium containing antibiotics and 10% FBS. Human microvascular endothelial cells (HMEC-1) were cultured in Medium 200 with low serum growth supplement (LSGS; Thermo Fisher Scientific) and 10% FBS. Huh-7, BHK-21, U937, and HMEC-1 cells were maintained in a humidified 5% CO_2_ atmosphere at 37°C. C6/36 cells were maintained at 28°C.

### Plaque assay

BHK-21 cells were plated into 12-well plates (8 × 10^4^ cells/well) and cultured in 5% RPMI in 5% CO_2_ overnight. Virus was serially diluted with RPMI medium and incubated with BHK-21 cells. After adsorption with serially diluted virus for 2 h, the medium was replaced with fresh DMEM containing 2% FBS and 0.8% methyl cellulose. At 5 d.p.i., the medium was removed, and the cells were fixed and stained with crystal violet solution consisting of 1% crystal violet, 0.64% NaCl, and 2% formalin overnight. The 12-well plates were washed with water and the virus titers were determined as PFU/ml.

### Generation of humanized monoclonal antibodies

Humanized mAbs 2E8 and 33D2 were obtained from DCB and Leadgene Biomedical, Inc., respectively, using CDR-grafting combined with hybridoma sequencing. In brief, the murine mAbs were humanized by grafting CDR onto variable regions of heavy chain (V_H_) and light chain (V_L_) frameworks of human IgG, then the sequences were cloned. Multiple cloned V_H_ and V_L_ regions were expressed in plasmid backbones, and the binding ability of resulting V_H_-V_L_ pairs expressed in mammalian expression system to target antigen were investigated [60]. Subsequently, the sequences of V_H_-V_L_ pairs with highest binding ability to antigen were cloned into human IgG1 backbone, because IgG1 has better effector functions such as CDC and ADCC [61,62].

### Binding ability of mAbs to NS1 as determined by ELISA

Full-length NS1 was coated on 96-well plates at 0.2 μg/well in phosphate-buffered saline (PBS) at 4°C overnight. The 96-well plates were blocked with 5% bovine serum albumin (BSA) in PBS at 37°C for 1 h, and then washed three times with 0.05% Tween 20 in PBS. Humanized 33D2 and 2E8 were diluted serially from 1000 ng/ml. The diluted mAbs were added into protein-coated wells and then incubated at room temperature for 1 h. After washing three times, peroxidase-conjugated anti-human IgG were added into each well and incubated for 1 h at room temperature. After washing three times, 3,3’,5,5’-Tetramethylbenzidine (TMB; Kementec) was added into each well and the absorbance at 450 nm was determined using an ELISA reader (EMax Microplate Reader; Molecular Devices).

### Flow cytometry

Huh7 cells were cultured in 6-well plates overnight at 37°C. On the following day, the cells were infected with four serotypes of DENV for 48 h. For surface staining, cells were stained with mouse or humanized anti-NS1 mAbs as well as mouse isotype control IgG2a or human isotype control IgG1 at 4°C for 1 h. After washing twice with staining buffer (2% FBS and 0.1% NaN_3_ in PBS), the samples were fixed with 4% paraformaldehyde in PBS on ice for 20 min. Then, the cells were washed with staining buffer followed by staining with FITC-conjugated anti-mouse/human IgG antibodies at 4°C for 30 min. For intracellular staining, cells were fixed with 4% paraformaldehyde in PBS on ice for 20 min. Then, these cells were washed with staining buffer followed by permeabilization with 0.1% saponin in staining buffer on ice for 20 min. After washing twice with staining buffer, these cells were incubated with mouse or humanized anti-NS1 mAbs as well as mouse isotype control IgG2a or human isotype control IgG1 at 4°C for 1 h. Cells were then washed with staining buffer and stained with FITC-conjugated anti-mouse/human IgG antibodies at 4°C for 30 min. The samples were analyzed by flow cytometry (CytoFLEX V2, Beckman Coulter System) with excitation set at 488 nm.

### DENV infection mouse model

The mouse model used in this study was originally established by Dr. Wu-Hsieh’s lab as reported [37–39]. Mice were intradermally (i.d.) inoculated with C6/36 control medium or DENV2 454009A (1 × 10^7^ PFU/mouse) at four different sites on the upper back. The mouse tail bleeding time and skin hemorrhage were determined on day 5. For passive immunization, mice were intraperitoneally (i.p.) administered with a single dose of mAbs at 1 d.p.i., or in one experiment at 1, 3 or 4 d.p.i. For another set of experiments, mice were challenged with DENV2 (1 × 10^7^ PFU/mouse) via intravenous (i.v.) route at day 0 and sacrificed at 3 d.p.i. Blood was collected and used for viremia and NS1 level measurements.

### Mouse tail bleeding time

Bleeding time of tail vein was performed by transecting the tail 3-mm from the tip. Blood droplets were collected on filter paper every 30 sec. Bleeding time was recorded when the blood spot was smaller than 0.1 mm in diameter.

### Hemorrhage quantification

After mice sacrifice, skin samples were photographed and the photographs were adjusted to the same image size. The grade of skin hemorrhage was digitally quantified by ImageJ software based on a published quantitative method [63] following image-processing using Photoshop 6.0. After conversion to 16-bit images and resetting as black and white photos, the total hemorrhage area was calculated and analyzed by Prism version 6.

### Fluorescent focus assay (FFA)

The sera from infected mice were serially diluted and incubated with BHK-21 cells for 2 h at 37°C. The monolayers were then overlaid with DMEM containing 0.8% methylcellulose and 2% FBS and incubated at 37°C for 48 h to 72 h. Virus foci were stained with anti-NS1 mAb followed by Alexa 488-conjugated goat anti-mouse IgG and then visualized by fluorescence microscopy (Olympus).

### NS1 quantitative ELISA

To quantify the NS1 levels, an in-house NS1 sandwich ELISA was performed. Briefly, 5 μg/ml anti-NS1 mAb 33D2 was coated onto 96-well plates at 4°C overnight. After blocking with 1% BSA in PBS for 1 h, mouse sera (1:5 dilutions) were co-incubated with 1 μg/ml biotin-conjugated anti-NS1 mAb 31B2 at room temperature for 1 h. HRP-labeled streptavidin solution (1:40) (R&D Systems) was added into wells for 30 min at room temperature. After washing three times with PBST (0.05% Tween 20 in PBS), TMB was added into the wells for color visualization. Following the addition of stop solution (2 N H_2_SO_4_), the absorbance was read at 450 nm using an EMax microplate reader.

### In vitro transwell permeability assay

To evaluate h33D2 in NS1-induced permeability change, HMEC-1 cells (3 × 10^5^/well) were seeded onto transwells for 48 h. Prior to an experiment, human IgG1 isotype control (BioXCell) or h33D2 were prepared in 100 μl M200 medium at indicated concentrations, followed by the addition of an equal volume of DENV1-4 NS1 (20 μg/ml; The Native Antigen Company) in M200 medium. After 30 min, media in the upper chambers were replaced with 200 μl mixtures and incubated for 6 h. Media were changed to 1 ml fresh M200 medium followed by the addition of 10 μl streptavidin-HRP (1:5000, 0.5 mg/ml; Leadgene Biomedical Inc.) and incubated for 30 min. Streptavidin-HRP in lower chambers were analyzed by using TMB. In brief, 20 μl of media from lower chambers were mixed with 100 μl TMB and incubated for 30 min at room temperature followed by adding 50 μl 1 N H_2_SO_4_ to stop the reaction. The absorbance at 450 nm was recorded and used to determine relative permeability.

### Complement-dependent cytolysis (CDC) assay

For CDC assay of the LALAPG variant, Huh-7 cells were used as target cells. Pooled normal human serum (NHS) was purchased from Innovative Research, Inc. and with or without heat-inactivation at 56°C for 30 min. Cell death was determined by using the LDH cytotoxicity colorimetric assay kit (BioVision). Experiments were based on 96-well assay. In brief, Huh-7 cells were seeded into wells of a 96-well culture plate at day 1 and infected at day 2. After 48 h, Huh-7 cells were replaced with 100 μl fresh medium (DMEM without phenol red; Thermo Fisher Scientific) with indicated human IgG1 isotype control (BioXCell), h33D2, and h33D2-LALAPG followed by the addition of 100 μl 10% NHS contained DMEM. Cell viability was analyzed after 4 h. To prepare positive control, 20 μl lysis buffer was added into a non-treated well. A non-infected well was used as negative control. Positive controls were calculated as mean values relative to the maximum LDH release. For the LDH assay, samples (20 μl) were mixed with 100 μl LDH reaction mix and incubated for 30 min at room temperature. Absorbance at 450 nm was recorded and the cytotoxicity percentage was calculated as follows:

% cytotoxicity = (Experimental value—NHS/Heat-inactivated NHS cell control—Negative control)

/ (Positive control—Negative control) x 100.

### Antibody-dependent cellular cytotoxicity (ADCC) assay

ADCC assays were performed by Leadgene Biomedical Inc. In brief, NK cells from peripheral blood mononuclear cells (PBMCs) (collected with ethical approval from the Institutional Review Board of National Cheng Kung University Hospital, No. A-ER-109-535, with written informed consent obtained from healthy volunteers) isolated by using Histopaque-1077 (Sigma-Aldrich) were expanded by ex vivo cultivation in IL-2/IL-15 (500 U/ml/1200 U/ml)-supplemented X-VIVOTM 15 medium (Lonza) for 21 days until use. The purity of NK cells was larger than 95%. Huh-7 cells (5 × 10^3^) were seeded onto 96-well culture plates at day 1 and infected with DENV1-4 at day 2. After 48 h, infected cells were used for ADCC experiments. Human IgG1 isotype control (BioXCell), h33D2, and h33D2-LALAPG were diluted in DMEM (without phenol red) in a 100 μl final volume. The ratio of NK cells (1.25 × 10^5^ cells in 100 μl serum-free RPMI 1640 medium) to Huh-7 cells (5 × 10^3^ cells) was 25:1. Effector cells were added after antibodies were incubated with cells for 15 min. The final experimental volume was 200 μl/well and incubated for additional 6 h. To determine LDH release, Huh-7 cells spontaneous LDH release control (Low control) were used as a reference. Positive control was prepared from 10% lysis buffer (LDH cytotoxicity colorimetric assay kit, BioVision) treated cells. The OD450 value of Huh-7 cells with various concentrations of antibodies and effector cells treatment were normalized with background LDH value. Cytotoxicity percentage was calculated as follows: % cytotoxicity = (Experimental value–NK background control—Low control) / (Positive control—Low control) x 100.

### Statistical analysis

All statistical analyses were performed using GraphPad Prism version 6.0 (GraphPad Software Inc.). The results were analyzed by one-way or two-way analysis of variance (ANOVA). *: p < 0.05, **: p < 0.01, ***: p < 0.001, ****: p < 0.0001 and ns indicates not significant for 95% two-tail confidence intervals.

## Supporting information

S1 Fig Humanized mAbs 2E8 and 33D2 are recognized by anti-human IgG but not by anti-mouse IgG. The binding ability of anti-mouse IgG (**A**) and anti-human IgG (**B**) to humanized 2E8-clone 69 (h2E8-69) and clone 70 (h2E8-70), humanized 33D2 (h33D2), isotype control human IgG1 (hIgG1), mouse 2E8 (m2E8), isotype control mouse IgG1 (mIgG1), mouse 33D2 (m33D2), and isotype control mouse IgG2a (mIgG2a) were determined by ELISA. A microtiter plate was coated with anti-mouse IgG (**A**) or anti-human IgG (**B**) followed by incubation with serial dilutions of h2E8-69, h2E8-70, h33D2, hIgG1, m2E8, mIgG1, m33D2, and mIgG2a. The absorbance at 450 nm was measured using a microplate reader.
(DOCX)

S2 Fig Stability analysis of humanized mAbs 2E8 and 33D2. (**A**) Humanized 2E8-clone 69 (h2E8-69) and clone 70 (h2E8-70), humanized 33D2 (h33D2), isotype control human IgG1 (hIgG1), mouse 2E8 (m2E8), isotype control mouse IgG1 (mIgG1), mouse 33D2 (m33D2), and isotype control mouse IgG2a (mIgG2a) were placed at 37°C for 7, 14, and 30 days. The protein concentrations were measured using a Nanodrop 2000. (**B**) Humanized 2E8-clone 69 (h2E8-69) and clone 70 (h2E8-70), humanized 33D2 (h33D2), and isotype control human IgG1 (hIgG1) were placed at 37°C for 7, 14, and 30 days. Then, the binding ability of h2E8-69, h2E8-70, h33D2 and hIgG1 were determined by ELISA as an indicator for functional stability. A microtiter plate was coated with DENV full length NS1 protein followed by incubation with serial dilutions of h2E8-69, h2E8-70, h33D2, and hIgG1. The absorbance at 450 nm was measured using a microplate reader. Two independent experiments were performed, and one set of representative results are shown.
(DOCX)

S3 Fig Anti-NS1 humanized mAbs do not induce ADE of DENV infection. The h2E8-69, h2E8-70, h33D2, isotype control hIgG1, anti-prM mAb 70.21, and control mouse IgG (cmIgG) (200 ng/ml) were preincubated with DENV2-454009A, and then inoculated into U937 cells. After 48 h incubation, the supernatants containing infectious DENV were collected and titrated by FFA. Anti-prM mAb 70.21 was used as positive control. Two-tailed Student’s *t*-test was used to determine statistical significance; *: p < 0.05, **: p < 0.01, ***: p < 0.001, ns indicates not significant. (n = 3 for each group) (ND: not detectable)
(DOCX)

S4 Fig Administration of mouse anti-NS1 mAb m33D2 and anti-E mAb m137-22 can shorten DENV-induced prolonged bleeding time. 1 × 10^7^ PFU/mouse DENV2-454009A or C6/36 control medium were inoculated i.d. into the upper back of *STAT1*^*-/-*^ mice. The mAbs m33D2, m137-22 or isotype control mIgG (50 μg/mouse) were injected i.p. four days after virus challenge. The tail bleeding time was determined on 5 d.p.i. (n = 2 for m137-22-treated group and n = 3 for other groups) *: p < 0.05, ****: p < 0.0001. Statistical significance was based on one-way ANOVA.
(DOCX)

S5 Fig The effects of DENV NS1 and TNF-α on endothelial permeability. DENV1-4 NS1- and TNF-α-induced HMEC-1 cell hyperpermeability was determined as described in the methods. All data are presented as the averages of triplicate cultures ± S.D. ns indicates not significant as compared with TNF-α group and analyzed by one-way ANOVA followed by Dunnett’s multiple comparison test.
(DOCX)

S6 Fig LALAPG mutation does not significantly affect the affinity and antibody clearance in vivo. (**A**) ELISA assays were performed using recombinant full length NS1 to assess the binding activities of h33D2 and h33D2-LALAPG. (**B**) The mice were i.p. injected with the mAbs h33D2, h33D2-LALAPG or isotype control hIgG1 (50 μg/mouse) and the sera were collected after 4 h, 2 days and 4 days post-adminstration. The anti-NS1 antibody levels of mouse sera were determined by ELISA.
(DOCX)

S7 Fig Effects of humanized anti-NS1 mAbs 33D2 and 33D2-LALAPG on DENV-induced prolonged bleeding time. 1 × 10^7^ PFU/mouse DENV2-454009A or C6/36 control medium were inoculated i.d. into the upper back of *STAT1*^*-/-*^ mice. The mAbs h33D2, h33D2-LALAPG or isotype control hIgG1 (50 μg/mouse) were injected i.p. four days after virus challenge. Tail bleeding time was determined on 5 d.p.i. (n = 4 for each group) *: p < 0.05, ****: p < 0.0001. Statistical significance was based on one-way ANOVA.
(DOCX)
