# Supplementary material for: Therapeutic efficacy of humanized monoclonal antibodies targeting dengue virus nonstructural protein 1 in the mouse model
Source: PLoS Pathog. 2022 Apr 29;18(4):e1010469. doi: 10.1371/journal.ppat.1010469 (PMC9053773; doi:10.1371/journal.ppat.1010469)
Supplement: S1 Fig — The binding ability of anti-mouse IgG (A) and anti-human IgG (B) to humanized 2E8-clone 69 (h2E8-69) and clone 70 (h2E8-70), humanized 33D2 (h33D2), isotype control human IgG1 (hIgG1), mouse 2E8 (m2E8), isotype control mouse IgG1 (mIgG1), mouse 33D2 (m33D2), and isotype control mouse IgG2a (mIgG2a) were determined by ELISA. A microtiter plate was coated with anti-mouse IgG (A) or anti-human IgG (B) followed by incubation with serial dilutions of h2E8-69, h2E8-70, h33D2, hIgG1, m2E8, mIgG1, m33D2, and mIgG2a. The absorbance at 450 nm was measured using a microplate reader. (DOCX) [file ppat.1010469.s001.docx]

**S1 Fig. Humanized mAbs 2E8 and 33D2 are recognized by anti-human IgG but not by anti-mouse IgG.** The binding ability of anti-mouse IgG (**A**) and anti-human IgG (**B**) to humanized 2E8-clone 69 (h2E8-69) and clone 70 (h2E8-70), humanized 33D2 (h33D2), isotype control human IgG1 (hIgG1), mouse 2E8 (m2E8), isotype control mouse IgG1 (mIgG1), mouse 33D2 (m33D2), and isotype control mouse IgG2a (mIgG2a) were determined by ELISA. A microtiter plate was coated with anti-mouse IgG (**A**) or anti-human IgG (**B**) followed by incubation with serial dilutions of h2E8-69, h2E8-70, h33D2, hIgG1, m2E8, mIgG1, m33D2, and mIgG2a. The absorbance at 450 nm was measured using a microplate reader.
