# Supplementary material for: Therapeutic efficacy of humanized monoclonal antibodies targeting dengue virus nonstructural protein 1 in the mouse model
Source: PLoS Pathog. 2022 Apr 29;18(4):e1010469. doi: 10.1371/journal.ppat.1010469 (PMC9053773; doi:10.1371/journal.ppat.1010469)
Supplement: S2 Fig — (A) Humanized 2E8-clone 69 (h2E8-69) and clone 70 (h2E8-70), humanized 33D2 (h33D2), isotype control human IgG1 (hIgG1), mouse 2E8 (m2E8), isotype control mouse IgG1 (mIgG1), mouse 33D2 (m33D2), and isotype control mouse IgG2a (mIgG2a) were placed at 37°C for 7, 14, and 30 days. The protein concentrations were measured using a Nanodrop 2000. (B) Humanized 2E8-clone 69 (h2E8-69) and clone 70 (h2E8-70), humanized 33D2 (h33D2), and isotype control human IgG1 (hIgG1) were placed at 37°C for 7, 14, and 30 days. Then, the binding ability of h2E8-69, h2E8-70, h33D2 and hIgG1 were determined by ELISA as an indicator for functional stability. A microtiter plate was coated with DENV full length NS1 protein followed by incubation with serial dilutions of h2E8-69, h2E8-70, h33D2, and hIgG1. The absorbance at 450 nm was measured using a microplate reader. Two independent experiments were performed, and one set of representative results are shown. (DOCX) [file ppat.1010469.s002.docx]

**S2 Fig. Stability analysis of humanized mAbs 2E8 and 33D2.** (**A**) Humanized 2E8-clone 69 (h2E8-69) and clone 70 (h2E8-70), humanized 33D2 (h33D2), isotype control human IgG1 (hIgG1), mouse 2E8 (m2E8), isotype control mouse IgG1 (mIgG1), mouse 33D2 (m33D2), and isotype control mouse IgG2a (mIgG2a) were placed at 37°C for 7, 14, and 30 days. The protein concentrations were measured using a Nanodrop 2000. (**B**) Humanized 2E8-clone 69 (h2E8-69) and clone 70 (h2E8-70), humanized 33D2 (h33D2), and isotype control human IgG1 (hIgG1) were placed at 37°C for 7, 14, and 30 days. Then, the binding ability of h2E8-69, h2E8-70, h33D2 and hIgG1 were determined by ELISA as an indicator for functional stability. A microtiter plate was coated with DENV full length NS1 protein followed by incubation with serial dilutions of h2E8-69, h2E8-70, h33D2, and hIgG1. The absorbance at 450 nm was measured using a microplate reader. Two independent experiments were performed, and one set of representative results are shown.
