# Supplementary material for: Therapeutic efficacy of humanized monoclonal antibodies targeting dengue virus nonstructural protein 1 in the mouse model
Source: PLoS Pathog. 2022 Apr 29;18(4):e1010469. doi: 10.1371/journal.ppat.1010469 (PMC9053773; doi:10.1371/journal.ppat.1010469)
Supplement: S3 Fig — The h2E8-69, h2E8-70, h33D2, isotype control hIgG1, anti-prM mAb 70.21, and control mouse IgG (cmIgG) (200 ng/ml) were preincubated with DENV2-454009A, and then inoculated into U937 cells. After 48 h incubation, the supernatants containing infectious DENV were collected and titrated by FFA. Anti-prM mAb 70.21 was used as positive control. Two-tailed Student’s t-test was used to determine statistical significance; *: p < 0.05, **: p < 0.01, ***: p < 0.001, ns indicates not significant. (n = 3 for each group) (ND: not detectable) (DOCX) [file ppat.1010469.s003.docx]

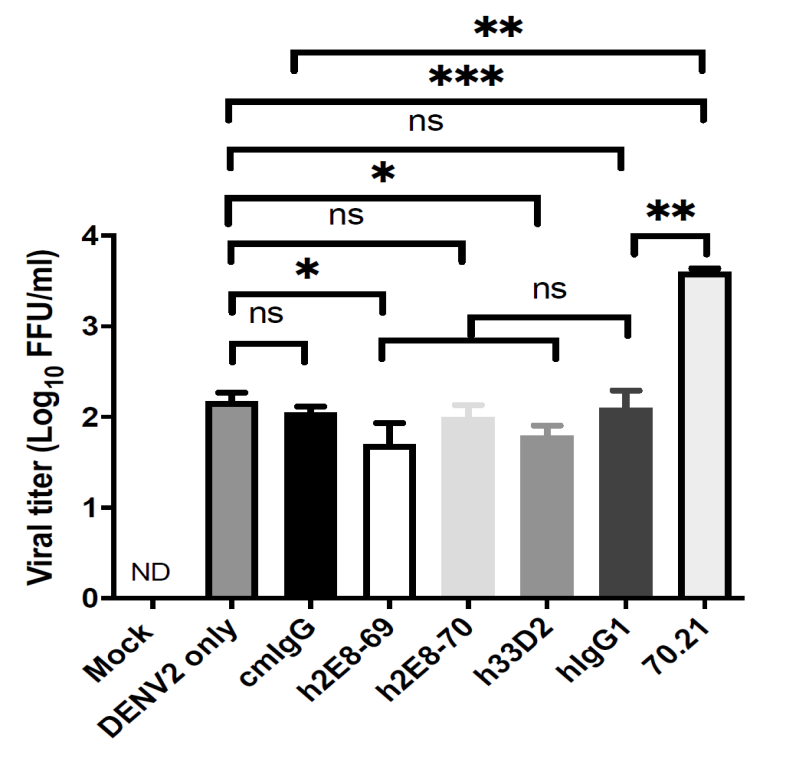


**S3 Fig. Anti-NS1 humanized mAbs do not induce ADE of DENV infection.** The h2E8-69, h2E8-70, h33D2, isotype control hIgG1, anti-prM mAb 70.21, and control mouse IgG (cmIgG) (200 ng/ml) were preincubated with DENV2-454009A, and then inoculated into U937 cells. After 48 h incubation, the supernatants containing infectious DENV were collected and titrated by FFA. Anti-prM mAb 70.21 was used as positive control. Two-tailed Student's *t*-test was used to determine statistical significance; *: p < 0.05, **: p < 0.01, ***: p < 0.001, ns indicates not significant. (n = 3 for each group) (ND: not detectable)
