# Supplementary material for: Therapeutic efficacy of humanized monoclonal antibodies targeting dengue virus nonstructural protein 1 in the mouse model
Source: PLoS Pathog. 2022 Apr 29;18(4):e1010469. doi: 10.1371/journal.ppat.1010469 (PMC9053773; doi:10.1371/journal.ppat.1010469)
Supplement: S4 Fig — 1 × 10^7^ PFU/mouse DENV2-454009A or C6/36 control medium were inoculated i.d. into the upper back of STAT1-/- mice. The mAbs m33D2, m137-22 or isotype control mIgG (50 μg/mouse) were injected i.p. four days after virus challenge. The tail bleeding time was determined on 5 d.p.i. (n = 2 for m137-22-treated group and n = 3 for other groups) *: p < 0.05, ****: p < 0.0001. Statistical significance was based on one-way ANOVA. (DOCX) [file ppat.1010469.s004.docx]

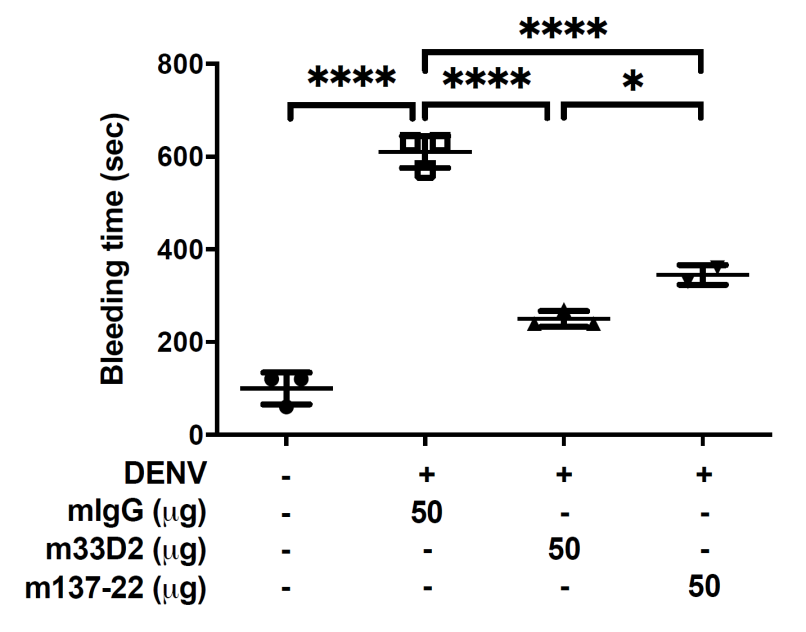


**S4 Fig. Administration of mouse anti-NS1 mAb m33D2 and anti-E mAb m137-22 can shorten DENV-induced prolonged bleeding time.** 1 × 10^7^ PFU/mouse DENV2-454009 or C6/36 control medium were inoculated i.d. into the upper back of *STAT1^-/-^* mice. The mAbs m33D2, m137-22 or isotype control mIgG (50 μg/mouse) were injected i.p. four days after virus challenge. The tail bleeding time was determined on 5 d.p.i. (n = 2 for m137-22-treated group and n = 3 for other groups) *: p < 0.05, ****: p < 0.0001. Statistical significance was based on one-way ANOVA.
