# Supplementary material for: Therapeutic efficacy of humanized monoclonal antibodies targeting dengue virus nonstructural protein 1 in the mouse model
Source: PLoS Pathog. 2022 Apr 29;18(4):e1010469. doi: 10.1371/journal.ppat.1010469 (PMC9053773; doi:10.1371/journal.ppat.1010469)
Supplement: S5 Fig — DENV1-4 NS1- and TNF-α-induced HMEC-1 cell hyperpermeability was determined as described in the methods. All data are presented as the averages of triplicate cultures ± S.D. ns indicates not significant as compared with TNF-α group and analyzed by one-way ANOVA followed by Dunnett’s multiple comparison test. (DOCX) [file ppat.1010469.s005.docx]

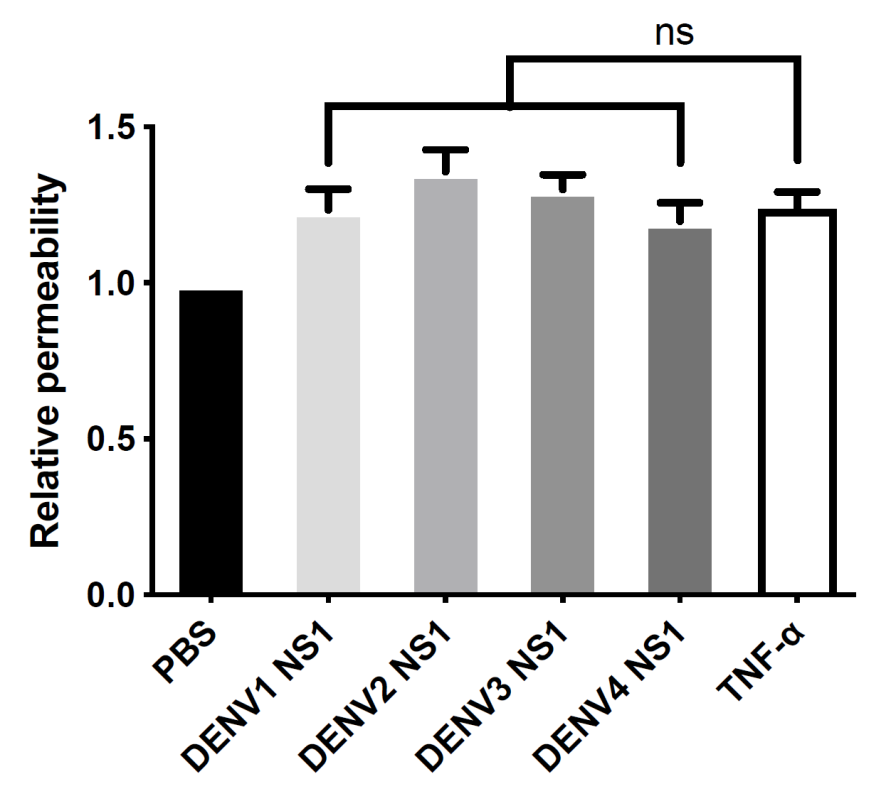


**S5 Fig. The effects of DENV NS1 and TNF-α on endothelial permeability.** DENV1-4 NS1- and TNF-α-induced HMEC-1 cell hyperpermeability was determined as described in the methods. All data are presented as the averages of triplicate cultures ± S.D. ns indicates not significant as compared with TNF-α group and analyzed by one-way ANOVA followed by Dunnett’s multiple comparison test.
