# Supplementary material for: Therapeutic efficacy of humanized monoclonal antibodies targeting dengue virus nonstructural protein 1 in the mouse model
Source: PLoS Pathog. 2022 Apr 29;18(4):e1010469. doi: 10.1371/journal.ppat.1010469 (PMC9053773; doi:10.1371/journal.ppat.1010469)
Supplement: S6 Fig — (A) ELISA assays were performed using recombinant full length NS1 to assess the binding activities of h33D2 and h33D2-LALAPG. (B) The mice were i.p. injected with the mAbs h33D2, h33D2-LALAPG or isotype control hIgG1 (50 μg/mouse) and the sera were collected after 4 h, 2 days and 4 days post-adminstration. The anti-NS1 antibody levels of mouse sera were determined by ELISA. (DOCX) [file ppat.1010469.s006.docx]

**
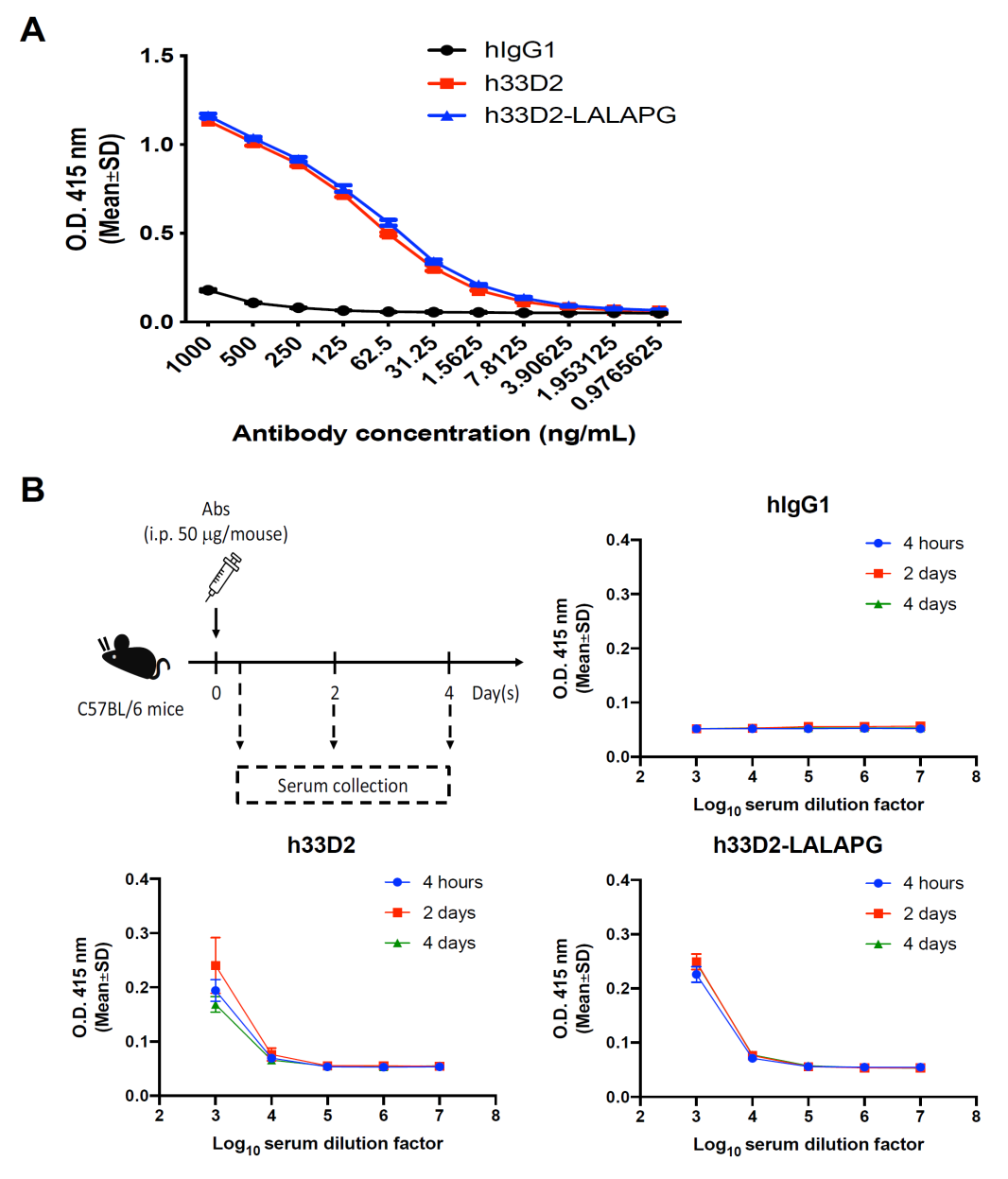
**

**S6 Fig. LALAPG mutation does not significantly affect the affinity and antibody clearance in vivo.** (**A**) ELISA assays were performed using recombinant full length NS1 to assess the binding activities of h33D2 and h33D2-LALAPG. (**B**) The mice were i.p. injected with the mAbs h33D2, h33D2-LALAPG or isotype control hIgG1 (50 μg/mouse) and the sera were collected after 4 h, 2 days and 4 days post-adminstration. The anti-NS1 antibody levels of mouse sera were determined by ELISA.
