# Supplementary material for: Therapeutic efficacy of humanized monoclonal antibodies targeting dengue virus nonstructural protein 1 in the mouse model
Source: PLoS Pathog. 2022 Apr 29;18(4):e1010469. doi: 10.1371/journal.ppat.1010469 (PMC9053773; doi:10.1371/journal.ppat.1010469)
Supplement: S7 Fig — 1 × 10^7^ PFU/mouse DENV2-454009A or C6/36 control medium were inoculated i.d. into the upper back of STAT1-/- mice. The mAbs h33D2, h33D2-LALAPG or isotype control hIgG1 (50 μg/mouse) were injected i.p. four days after virus challenge. Tail bleeding time was determined on 5 d.p.i. (n = 4 for each group) *: p < 0.05, ****: p < 0.0001. Statistical significance was based on one-way ANOVA. (DOCX) [file ppat.1010469.s007.docx]

**
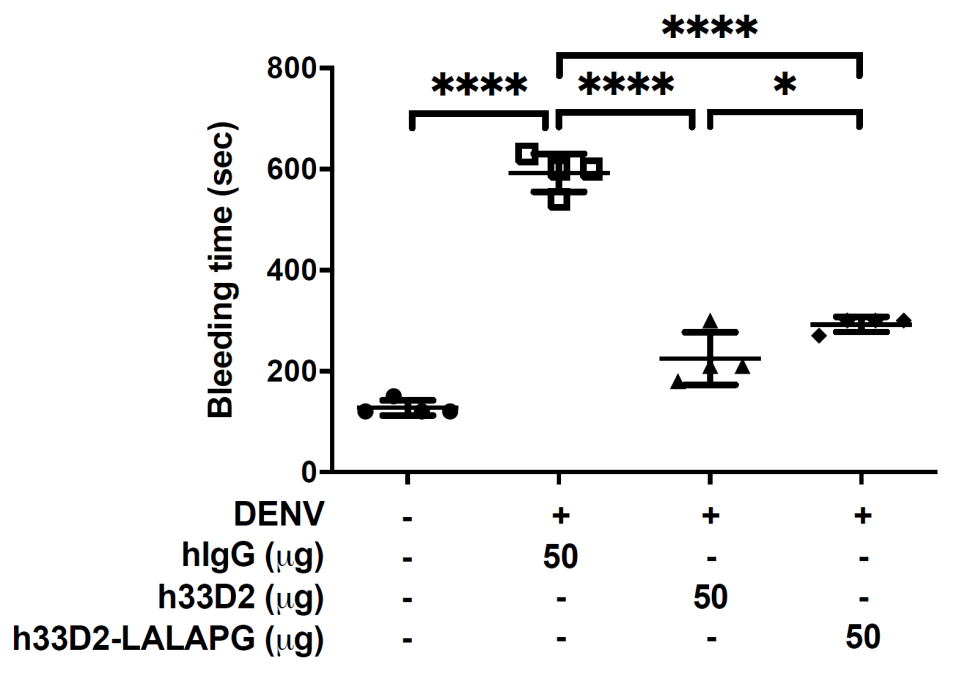
**

**S7 Fig. Effects of humanized anti-NS1 mAbs 33D2 and 33D2-LALAPG on DENV-induced prolonged bleeding time.** 1 × 10^7^ PFU/mouse DENV2-454009 or C6/36 control medium were inoculated i.d. into the upper back of *STAT1^-/-^* mice. The mAbs h33D2, h33D2-LALAPG or isotype control hIgG1 (50 μg/mouse) were injected i.p. four days after virus challenge. Tail bleeding time was determined on 5 d.p.i. (n = 4 for each group) *: p < 0.05, ****: p < 0.0001. Statistical significance was based on one-way ANOVA.
